# Renal cell carcinoma with metastasis to the pancreas: a model for oligometastasis, oligoprogression and metastatic organotropism

**DOI:** 10.1007/s10585-025-10359-w

**Published:** 2025-07-03

**Authors:** Kjetil Søreide, Elen Martine Hauge, Maria Nyre Vigmostad

**Affiliations:** 1 Department of Gastrointestinal Surgery, Stavanger University Hospital, Stavanger, Norway; 2 Department of Clinical Medicine, University of Bergen, Bergen, Norway; 3 Gastrointestinal Translational Research Unit, Lab for Molecular medicine, Stavanger University Hospital, Stavanger, Norway; 4 Department of Surgery, Section of Urology, Stavanger University Hospital, Stavanger, Norway; 5 Department of Oncology, Stavanger University Hospital, Stavanger, Norway

**Keywords:** Metastasis, Renal cell carcinoma, Pancreatic metastasis, Organotropism, Oligometastasis, Oligoprogression

## Abstract

Metastatic cancer has been considered uniformly fatal in the past with very poor outcomes for most cancer sites. However, novel systemic and targeted therapies have rendered unique responses with longer survival across several cancer types and metastatic sites. In addition, improved surgical experience and safety with good outcomes has made metastasectomy as an alternative curative-intent treatment across multiple organ sites. The pancreas is an uncommon site for metastasis, even if >30 different primary tumor entities have been described to metastasize to the pancreas. More than half of all resected metastasis in the pancreas are from renal cell carcinoma (RCC). RCC demonstrates a particular capacity to metastasize to nearly any site in the body—including uncommon sites like the tongue, salivary glands, spleen, testes, and pancreas—and, have remarkable plasticity and specific molecular trajectories with clinical implications. Cancer cells have a propensity to metastasize to specific organ sites, such as the lungs, liver or skeleton, called “organotropism” and the inherent tumor biology as well as the concept of ‘oligometastatic’ disease is still controversial and conflicting. Pancreatic metastasis has a very different biology from other RCC metastatic sites. Clinical observations suggest an indolent biology that warrants further investigation. Survival times are very long and approaching up to 10 years in recent series. In this paper we discuss the specific situation of pancreatic metastasis from RCC, the relation to oligometastasis and organotropism and how this can be viewed as a model to better understand cancer biology.

## Introduction

Metastatic cancer has been considered uniformly fatal in the past with very poor outcomes for most cancer sites. However, novel systemic and targeted therapies have rendered unique responses with longer survival across several cancer types and metastatic sites. In addition, improved surgical experience and safety with good outcomes has made metastasectomy as an alternative curative-intent treatment across multiple organ sites—most often reported for metastasectomy in liver and lungs—but also including the pancreas. While cancers developing in the pancreatic gland (i.e. pancreatic ductal adenocarcinoma) are extraordinarily aggressive and treatment resistant, the rare occurrence of secondary tumors in the pancreatic gland seem to behave much more favorably, in particular so metastasis from renal cell carcinomas (RCC) that are reported with very long median survival times after resection [1–5]. In a study of two different cohorts of metastatic RCC and metastasis at several sites, the median overall survival was reported at 90 and 121 months, respectively [6]. A more recent nested case control study of patients with liver or pancreatic metastasis reported a 5-year survival in mRCC at 75% of those who had surgery and just over 50% for non-operated patients [7]. Overall, the 5-year survival rate in mRRC has increased over the decades from < 10% to over 30% in nationwide series [8] and depending on type of treatment provided in trials [9].

The International Metastatic RCC database Consortium (IMDC) score is used for predicting prognosis in patients with metastatic RCC with the estimated prognosis varying from 42 months favorable risk groups to only 8 months in the poor risk group [9]. The treatment landscape of metastatic RCC has evolved rapidly the last decade with the introduction of immunotherapy combinations that has led to an increased overall survival compared to the previous standard treatment with tyrosine kinase inhibitor sunitinib [10–12].

Globally, RCC represents the 14th most frequently diagnosed cancer, with more than 400 000 new cases reported in 2020 [10] and a continued increase in age-standardized incidence rates around the world [13]. The 5-year overall survival for all RCC in Norway is reported at > 80%. With an increasing incidence of RCC diagnosed every year, one may expect a larger number of patients that will develop metastasis even after radical treatment with the overall risk of metastases in RCC being 30% and upward, depending on stage and associated risk features.

### The pancreas as a metastatic site

**Fig. 1:**
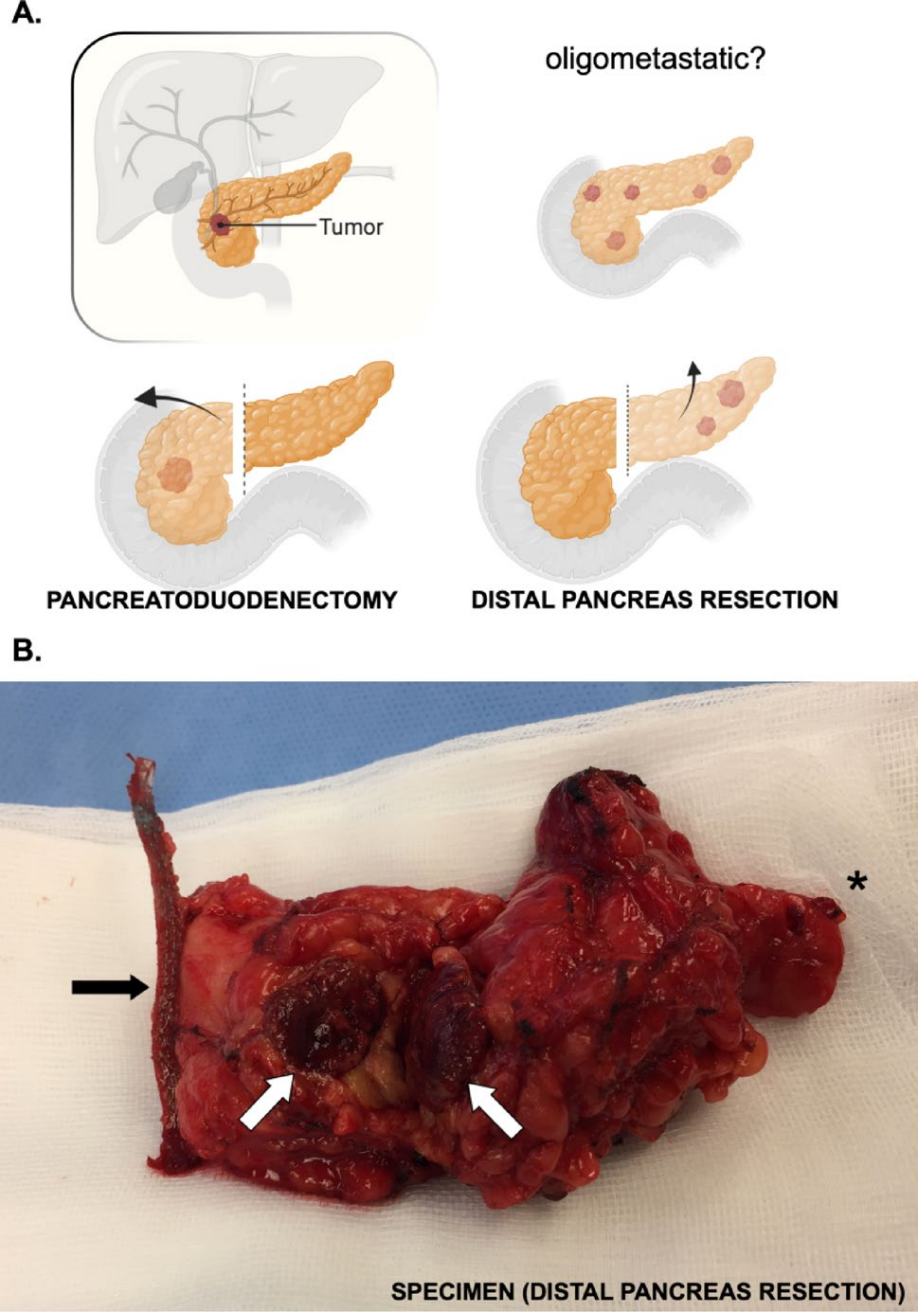
Pancreatic surgery for metastatic renal cell carcinoma. Legend: **A** consideration of type of surgery influences decision to operate. Oligometastasis is poorly defined, but non-surgical consensus work suggests up to 5 lesions. Not shown are other options, such as total pancreatectomy or enucleation, with their own associated morbidity and long-term effects. **B** Distal pancreatectomy for single metastasis from renal cell carcinoma. Black arrow indicates staple line for transection towards the pancreatic body/head. White arrows indicate lesion in pancreatic tail, depicting metastasis from RCC. Asterisks indicate tail towards distal spleen, a spleen-preserving resection was performed

In patients with metastasis to the pancreas, the most common primary cancer is renal cell carcinoma (RCC), described already more than 6 decades ago [14]. More than 30 different primary sites of origin have been described in series resecting metastases to the pancreas [15–17]. For all metastasis to the pancreas that are resected (Fig. 1), RCC is the primary tumor of origin in 55–60% of all patients across surgical series [15, 17–19]. This despite RCC being far outnumbered by more common tumors—including prostate cancer, breast cancer, lung cancer and colorectal cancer—that are only rarely if ever reported to metastasize to the pancreas.

**Fig. 2:**
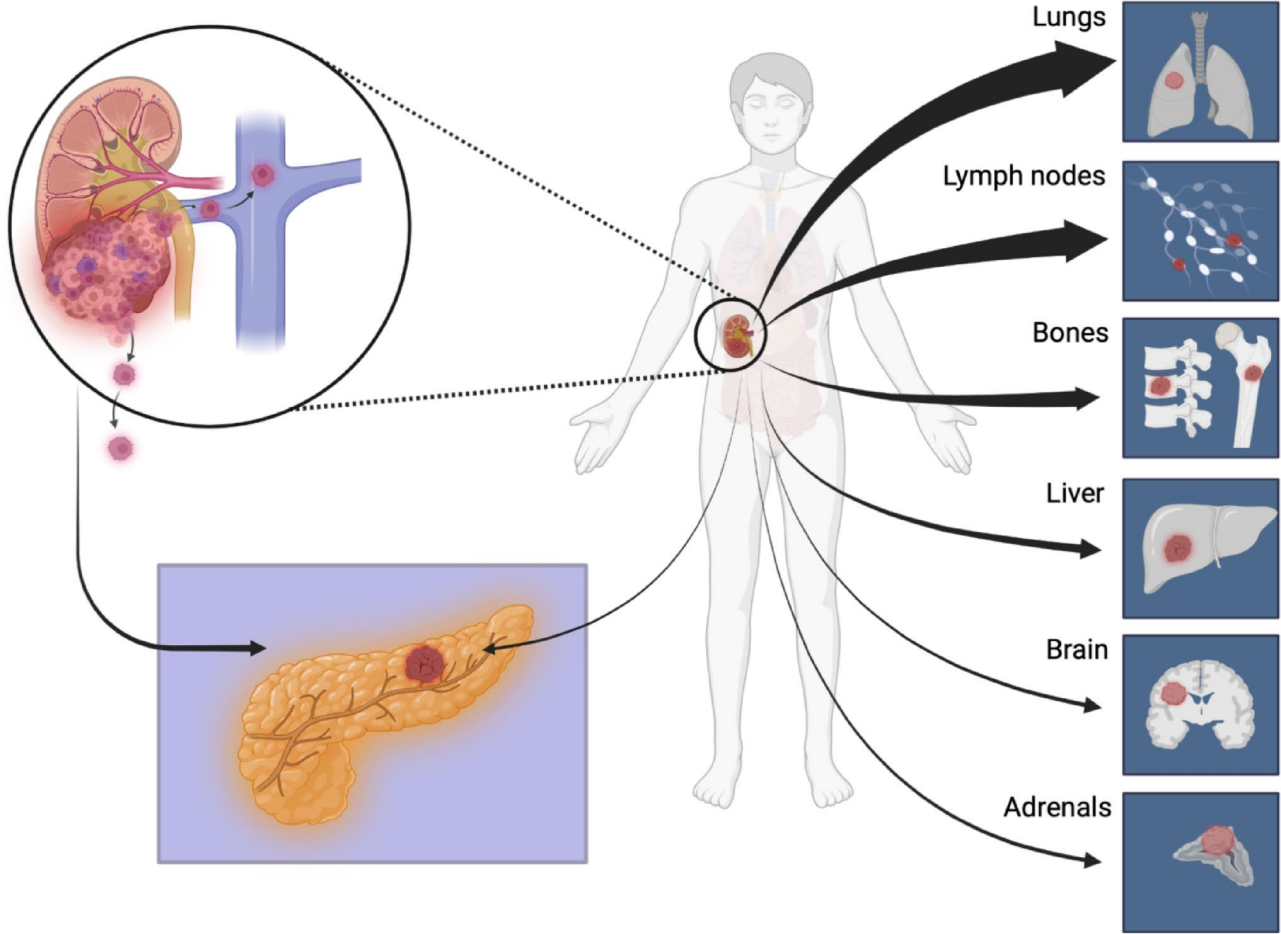
Common metastatic sites in renal cell carcinoma. Legend: Common sites of metastasis involve the lung in 70%, lymph nodes in 45%, followed by bone in 32%, liver 18%, adrenal glands in 10%, and brain in 8%, and with some 5% in the pancreas

Even if RCC is known to have a predilection to metastasize to the pancreas, the most common sites of metastasis involves the lung (70%), lymph nodes (45%), bone (32%), liver (18%), adrenal glands (10%), and brain (8%), with the pancreas only being the 7th most common organ site (Fig. 2) of disease spread from RCC [20]. In one Italian study of 2283 patients with metastatic RCC, some 103 (4.5%) had metastasis to the pancreas [21]. This is in line with a large study of over 10.000 patients from the International mRCC Database Consortium [20], that found 5% of clear cell RCC metastasis to the pancreas, and a much lower frequency in papillary or chromophobe RCC (at 1–2%).

The role of surgery in metastatic RCC is debated [21–24]. Upfront cytoreductive nephrectomy in synchronous metastatic RCC used to be standard of care. However, after the findings reported from the Carmena and Surtime studies is now considered only for selected patients in good and sometimes intermediate risk groups, dependent on treatment response [23, 24]. Ongoing studies aims to evaluate the effect of deferred cytoreductive nephrectomy for intermediate risk patients treated in the immunotherapy era [25]. Other local treatments in RCC that can be considered for patients with low metastatic burden like metastasectomy, stereotactic radiosurgery and radiotherapy, lacks robust, prospective data [26, 27]. Although associated with favorable outcomes, most likely patient selection is key. With adjuvant immunotherapy recently demonstrating survival benefit for patients that have had a complete metastasectomy with no evidence of disease after surgery, multimodal therapy should now be considered in combinations (an overview is given in Table 1), including the option for surgery for specific metastatic locations [6, 10, 28]. Consequently, controversies to the current role of surgery needs to be revisited as further options for systemic and locoregional therapy have emerged [29]. However, data indicates that biological differences exist according to metastatic site [30] and molecular subtypes [31], such that either locoregional or systemic therapy may not have an expected similar effect across metastatic sites, with less effective therapy for certain organ metastasis and molecular subtypes than others [31]. Thus, even if more therapeutic options become available and even if metastasis to the pancreas behave favorably, these lesions may be less responsive to targeted drugs or immunotherapy and, as such, suggest surgical removal as a better option in suitable candidates.

**Table 1:** Treatment strategies in metastatic renal cell carcinoma (mRCC)

Adjuvant immunotherapy^a^	Offer when no evidence of disease (NED) after resection of oligometastatic sites of clear cell RCC ≤ 1 year from nephrectomy		
Local treatment^b^	Offer ablative therapy, including metastasectomy, when complete resection is possible Offer radiotherapy for metastasis for local control and symptom relief		
	**IMDC prognostic groups**^c^		
	**Favourable**	**Intermediate**	**Poor**
Cytoreductive nephrectomy^d^	*Immediate*: Selected patients that do not need immediate systemic treatment When complete local treatment is achievable *Delayed*: Selected patients with clinical benefit from systemic treatment		Not indicated
Systemic treatment in first line setting of clear cell RCC according to ESMO guidelines^e*^	Cabozantinib/ Nivolumab Axitinib/ Pembrolizumab Lenvatinib/ Pembrolizumab Nivolumab/ Ipilimumab Sunitinib Pazopanib Tivozanib		Cabozantinib/ Nivolumab Axitinib/ Pembrolizumab Lenvatinib/ Pembrolizumab Nivolumab/ Ipilimumab For patients who cannot receive immune checkpoint inhibitors: Cabozantinib Sunitinib Pazopanib

^a^Choueiri, T.K., et al. Overall Survival with Adjuvant Pembrolizumab in Renal-Cell Carcinoma. N Engl J Med, 2024. 390: 1359

^b^Dabestani S, Marconi L, Hofmann F, et al. Local treatment for metastases of renal cell carcinoma: a systematic review. Lancet Oncol. 2014;15(12):e549-e561

^c^Heng, D.Y., et al. Prognostic factors for overall survival in patients with metastatic renal cell carcinoma treated with vascular endothelial growth factor-targeted agents: results from a large, multicenter study. J Clin Oncol, 2009. 27: 5794

^d^Mejean A, Ravaud A, Thezenas S, et al. Sunitinib alone or after nephrectomy in metastatic renal-cell carcinoma. *N Engl J Med*. 2018;379(5):417–427

^e^Renal cell carcinoma: ESMO Clinical Practice Guideline for diagnosis, treatment and follow-up^☆^ Powles, T. et al. Annals of Oncology, Volume 35, Issue 8, 692– 706

*Different treatment strategies applies for variant histologies

### Increasing cohorts with oligometastatic and oligoprogressive disease

Large data cohorts demonstrate an increasing use of singe- and multi-agent therapies as well as immunotherapy across several tumor types [32]. Surgery may thus become an option for those with oligometastatic or oligoprogressive disease, for which long-term systemic control may be achieved and even aimed at curative-intent surgery in some patients.

Notably, this relies on the current ability to understand the cancer biology and availability of multimodal therapy and appropriate treatment sequencing [26]. Available treatment options and lines of treatments offered across tumor types continues to increase, and with increased survival, more patients have stable (albeit uncurable) disease over longer time intervals. Oligometastatic disease was defined only 2 decades ago [33], yet has still to be properly defined beyond descriptive data and empirical evidence [34]. Huge variation and heterogeneity exist in the clinical presentation and behavior, as well as in the treatment offered [6]. Some patients experience single progressive metastasis (oligoprogression) that could be offered resection. Others may have undergone initial curative surgery but develop limited metastatic disease (oligometastasis) that may be available for technical resectability. However, as the concept of oligometastasis is evolving [35], there is controversy to the definitions across cancer locations and the role of available treatment modalities. For example, in bladder cancer the maximum number of lesions was defined as 3, while in renal cancer up to 5 lesions are considered ‘oligometastatic’ [36]. For which patients and when surgery should be considered may be controversial [37], such as also discussed for melanoma [38] and RCC [37], and even in select patient with lung cancer to the pancreas [39]. In addition to the somewhat controversial concept of oligometastatic disease definition per se [35], some metastatic locations are rather rare, such as metastasis to the pancreas, and hence the available data for treatment decisions is rather limited. Of note, the existing definition of oligometastasis is based on a European Society for Radiotherapy and Oncology and European Organization for Research and Treatment of Cancer consensus recommendation [35] without any inclusion of surgeons for consideration to the definitions. Furthermore, a similar consensus on treatment of oligometastatic disease in RCC did not reach consensus on the use of SABR in treatment of isolated metastasis to the pancreas [27]. Hence, surgery may have a complementary role to the available systemic treatment options [29], as the treatment options and selection criteria have become more complex [6].

### Organotropism—a predilection to spread to a distant organ

Cancer cells have a propensity to metastasize to specific organ sites, such as the lungs, liver or skeleton, called “organotropism” [40] that are driven by complex biological mechanisms [41]. The current understanding of metastatic organotropism [42] and the inherent tumor biology as well as the concept of ‘oligometastatic’ disease is still controversial and conflicting. The clear cell histological subtype in RCC makes up > 80% of all RCC and demonstrates a particular capacity to metastasize to nearly any site in the body—including uncommon sites like the tongue, salivary glands, spleen, testes, and pancreas—and, have remarkable plasticity and specific molecular trajectories with clinical implications [43]. Why this specific cancer predominates in pancreatic metastasis and has such a favorable prognosis is currently largely unknown. Indeed, the pancreas it is not a typical organ to metastasize to, nor is it the most frequent site of metastasis even in metastatic RCC. Indeed, gastrointestinal metastasis from RCC is overall rare and considered to have an overall poor prognosis [44]. Hence, the rarity of metastatis to the pancreas, yet the rather favorable prognosis of metastasis to this site from RCC warrants further research into the complexity of cancer biology.

The anatomical relation from the left kidney to the pancreatic gland should stipulate a seed-over theory, yet metastasis to the pancreatic gland is equally common from RCC in both the left and the right kidney and reported with near similar rates and spread across the pancreatic gland. Thus, this suggest a hematogenous spread, rather than local skip metastasis.

An important part in better knowledge of specific organotropism is to understand the pre-metastatic niche [45]. The pre-metastatic nice is a complex theme that is best understood from research developed over the past recent years, but largely in other sites and for other tumors [46], and involves epithelial-mesenchymal transition, immunosuppression, extracellular matrix remodeling, metabolic reprogramming, vascular permeability and angiogenesis [46, 47]. For metastatic RCC, a comprehensive review of the current knowledge of the pre-metastatic nice was recently published [48]. The pre-metastatic niche involves a complexity of mechanisms, with potential for therapeutic interventions of these mechanisms. However, due to its rarity, it is nearly impossible to consider investigation of the pre-metastatic niche in the pancreatic gland, but hematogenous spread along molecular mechanisms as proposed for other metastasis is a conceivable route to the pancreatic gland [49].

It is not clear from the available literature if the RCC-specific organotropism can be translated to other primary cancer types. Hence, one would currently have to rely on and extrapolate results from other disease-specific metastatic sites and make an evaluation for every patient regarding the site of the primary, the time from initial treatment of tumor of origin, whether the disease represents true ‘oligometastatic’ disease (i.e. up to 5 lesions defined as oligometastasis in RCC) or may indeed be more aggressive, widespread disseminated cancer (Fig. 1). Further translational evidence coupling primary organ site with the mechanisms related to organotropism in pancreatic metastasis (and, compared to other sites of metastasis) need to follow to demonstrate biological plausibility for a therapeutic rationale that currently rests on observation and is unlikely to arrive at data from randomized trials [50].

### Survival after surgery for metastasis to the pancreas

Even if metastases to the pancreas are distributed equally across the gland, with no particular predilection, distal pancreatic resections are most commonly reported. The fact that surgery is performed more often as a distal pancreatectomy [15] (removal of the pancreatic tail; Fig. 1) compared to the pancreatoduodenectomy (also called a Whipple’s procedures; or, removal of the pancreatic head with adjacent organs) is more likely attributed to the difference in morbidity (and even mortality) between these two procedures, with much greater risks for both mortality and morbidity with pancreatoduodenectomy). A distal pancreatic resection, now frequently done as a minimal-invasive procedure [51, 52] with less morbidity and faster recovery, may be more easily entertained and discussed with the patient, compared to the pancreatoduodenectomy that comes with both a higher mortality risk and associated morbidity burden (Fig. 1AB).

Median overall survival after surgery for pancreatic RCC-metastasis is increasing in the reported literature and now approaching and even surpassing 10 years median survival after pancreatectomy in several series [2, 4, 53, 54]. Arguments have been put forward that the overall survival does not differ significantly when compared to patients treated with TKI or immunotherapy alone [6, 21]. However, the numerical survival time difference was 17 months in favor of surgery [21]. In many other cancer outcome comparisons, an overall survival time difference of 17 months would be considered a remarkable difference in most oncological trials. Furthermore, the number of patients who live with no disease is higher after surgery, even if long-term cure cannot be assured. In a multicenter study from 40 institutions in Spain, the disease-free survival rate at 1, 3, and 5 years was 73%, 49%, and 35%, respectively [53]. This is in line with other contemporary data, showing 5-year survival at 60% and 1 in every 3 patients being disease free at 5 years after surgery for RCC metastasis to the pancreas [2–4, 53]. Adjuvant immunotherapy with pembrolizumab has showed a survival benefit for patients with no evidence of disease after surgery [28]. Further follow up and studies are needed to further explore this treatment options in patients with pancreatic metastasis considering both the long median survival for this particular subgroup of patients, and the morbidities associated with both surgery and systemic therapy. Importantly, around 10% of patients may be subject to repeat pancreatic resection (likely when the first operation was a minor distal pancreatectomy or, done as enucleations) in the course of the disease, with no difference in complication rates reported and with good survival outcomes reported [55]. Hence, surgery offers good outcomes, with long disease-free intervals and potential for long-term survival in selected patients.

### The favorable cancer biology is poorly understood

**Fig. 3:**
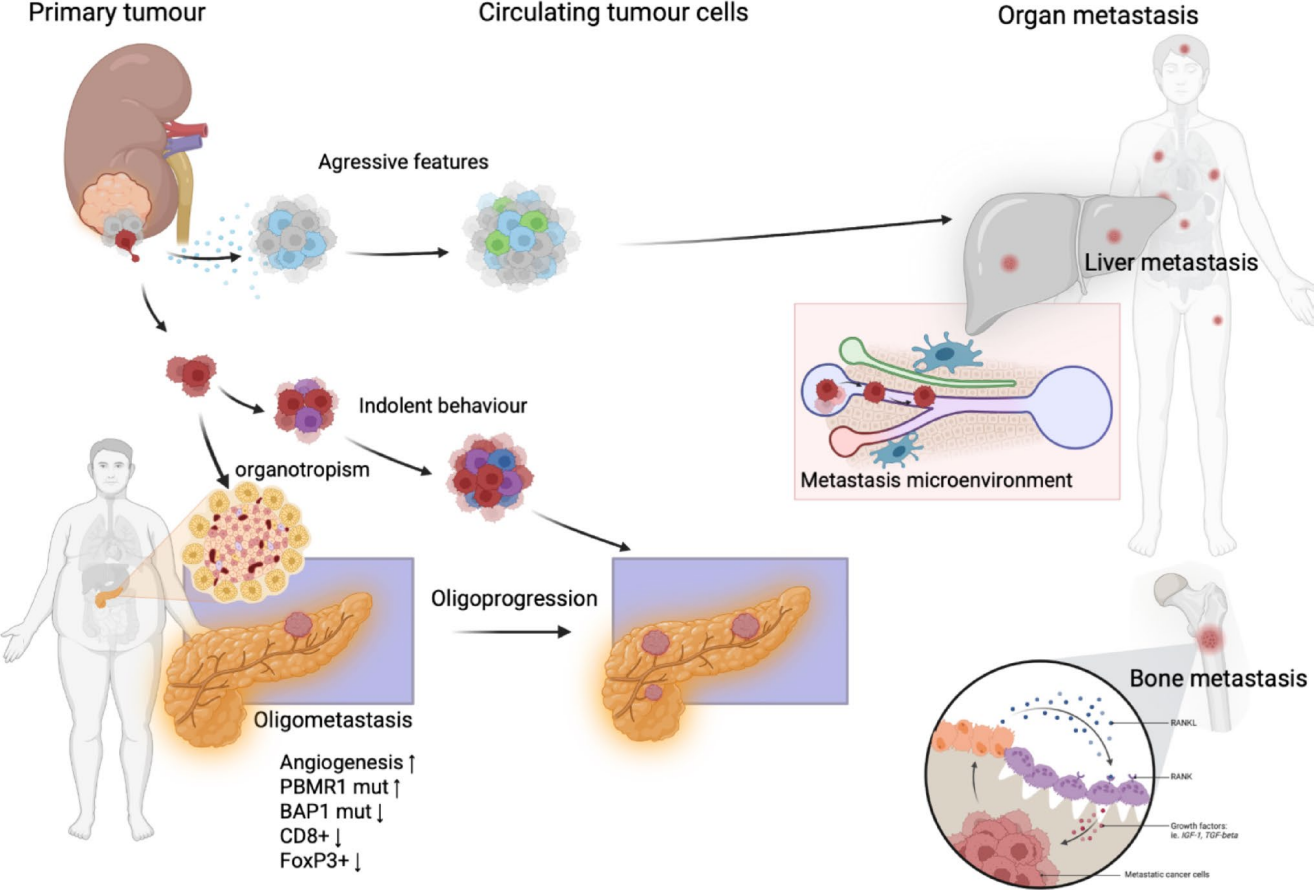
Variation in tumor biology, organotropism and cancer biology in RCC metastasis. Legend: simple illustration of heterogeneity in cellular plasticity, organotropism and cell behavior, with further details in the text

RCC has a remarkable cellular plasticity [43], which is essential for the ability to metastasize [56] and, may be reflected in its ability to metastasize to several sites and with different biological aggressiveness in cancer behavior [43]. Pancreatic metastasis has a very different biology from other RCC metastatic sites (Fig. 3), as observed clinically with an indolent biology and very long-term survival [57].

Indeed, the remarkable good prognosis related to RCC that metastasize to the pancreas with data supporting the role for surgical resection, may be further substantiated by data investigating the underlying cancer biology [58–60]. Studies investigating transcriptomic and genomic alterations find molecular alterations (i.e. such as in the PI3K/mTOR pathway amenable to tumor kinase inhibitors [59]) and genomic upregulations that are associated with a favorable outcome, such as frequent PBRM1 mutations [60], 3p loss, and 5q amplification. Also, a lower frequency of aggressive features such as BAP1 mutations and loss of 9p, 14q, and 4q are found, including pathways of angiogenesis [58]. An RNA-seq study based on few samples of different metastatic sites, including to the pancreas, found alterations in the metabolic signaling and activation of PAX8-myc signaling [58]. Most cells captured in single-cell RNA-seq were epithelial and malignant cells, T cells and macrophages, with reduced and disorganized fibroblasts [58], which might contribute to the downregulated and remodeling of extracellular matrix in the tumor microenvironment. Further, effects of hypoxia, an inflammatory tumor microenvironment and a metabolic reprogramming was found in the analysis, which allowed for generation of hypothesis but no confirmatory data. Other studies have similarly demonstrated considerable heterogeneity in the metastasis of RCC [61, 62] of which some could have impact on therapy and outcomes.

In a study of 31 patients with RCC and metastasis to the pancreas [60], the investigators found specific molecular traits that could explain the observed favorable outcome. Matched primary RCCs with pancreatic metastasis exhibited frequent PBRM1 mutations [60], 3p loss, and 5q amplification, together with a lower frequency of aggressive features such as BAP1 mutations and loss of 9p, 14q, and 4q [60]. Gene expression analyses revealed constrained evolution with remarkable uniformity, reduced effector T cell gene signatures, and increased angiogenesis. Hence, the pancreas metastasis is characterized by an indolent biology, heightened angiogenesis, and an uninflamed stroma, likely underlying its good prognosis, sensitivity to antiangiogenic therapies, and refractoriness to ICI [60]. These data suggest that metastatic organotropism may be an indicator of a particular biology with prognostic and treatment implications [57, 58, 60]. In a further study, a low intratumoral density of CD8 + and FOXP3 + lymphocytes was found in the pancreas specimens with RCC metastasis, compared to other tumors [30]. The low counts of CD8 + and FOXP3 + lymphocytes may reflect less aggressive features of clear-cell RCC with pancreatic metastasis that may result in a more favorable patient prognosis.

## Conclusion

Taken together, the results suggest specific molecular alterations in metastasis to the pancreas from RCC, that are associated with angiogenesis, tumor immune infiltration and metabolic reprograming that is associated with a favorable biological behavior and may partly explain the indolent nature of RCC cell-behavior in this particular metastatic site. Better description of the definitions and understanding of the relevant cancer biology of both the oligometastasis and oligoprogression is needed. Consensus on oligometastasis and oligoprogression should preferably include a wider multidisciplinary panel, including surgical oncologists. Aim should be to further refine the role of various treatment options as novel drug targets and locoregional options become available for this rare metastatic site [34, 37]. Surgery seems to offer very good long-term outcome in selected patients for patients with RCC metastasis to the pancreas, in particular with long disease-free interval before metastasis occur (up to 10 years) and with absence of symptoms. Availability of systemic therapies, targeted drugs and immunotherapy in addition to radiation therapy and stereotactic ablation body radiotherapy requires comprehensive team discussion to arrive at best treatment option for each patient.

## Data Availability

No datasets were generated or analysed during the current study.
